# Sugar Modification of Wall Teichoic Acids Determines Serotype-Dependent Strong Biofilm Production in Listeria monocytogenes

**DOI:** 10.1128/spectrum.02769-22

**Published:** 2022-10-03

**Authors:** Myungseo Park, Jinshil Kim, Liz Horn, Jisun Haan, Ali Strickland, Victoria Lappi, David Boxrud, Craig Hedberg, Sangryeol Ryu, Byeonghwa Jeon

**Affiliations:** a Division of Environmental Health Sciences, School of Public Health, University of Minnesota, Minneapolis, Minnesota, USA; b Department of Food and Animal Biotechnology, Research Institute of Agriculture and Life Sciences, and Center for Food and Bioconvergence, Seoul National University, Seoul, Republic of Korea; c Department of Agricultural Biotechnology, Seoul National University, Seoul, Republic of Korea; d Public Health Laboratory, Minnesota Department of Health, Saint Paul, Minnesota, USA; University of Florida

**Keywords:** *Listeria*, biofilms, serotype, cell wall, rhamnosylation

## Abstract

Biofilm production is responsible for persistent food contamination by Listeria monocytogenes, threatening food safety and public health. Human infection and food contamination with L. monocytogenes are caused primarily by serotypes 1/2a, 1/2b, and 4b. However, the association of biofilm production with phylogenic lineage and serotype has not yet been fully understood. In this study, we measured the levels of biofilm production in 98 clinical strains of L. monocytogenes at 37°C, 25°C, and 4°C. The phylogenetic clusters grouped by core genome multilocus sequence typing (cgMLST) exhibited association between biofilm production and phylogenetic lineage and serotype. Whereas clusters 1 and 3 consisting of serotype 4b strains exhibited weak biofilm production, clusters 2 (serotype 1/2b) and 4 (serotype 1/2a) were composed of strong biofilm formers. Particularly, cluster 2 (serotype 1/2b) strains exhibited the highest levels of biofilm production at 37°C, and the levels of biofilm production of cluster 4 (serotype 1/2a) strains were significantly elevated at all tested temperatures. Pan-genome analysis identified 22 genes unique to strong biofilm producers, most of which are related to the synthesis and modification of teichoic acids. Notably, a knockout mutation of the *rml* genes related to the modification of wall teichoic acids with l-rhamnose, which is specific to serogroup 1/2, significantly reduced the level of biofilm production by preventing biofilm maturation. Here, the results of our study show that biofilm production in L. monocytogenes is related to phylogeny and serotype and that the modification of wall teichoic acids with l-rhamnose is responsible for serotype-specific strong biofilm formation in L. monocytogenes.

**IMPORTANCE** Biofilm formation on the surface of foods or food-processing facilities by L. monocytogenes is a serious food safety concern. Here, our data demonstrate that the level of biofilm production differs among serotypes 1/2a, 1/2b, and 4b depending on the temperature. Furthermore, sugar decoration of bacterial cell walls with l-rhamnose is responsible for strong biofilm production in serotypes 1/2a and 1/2b, commonly isolated from foods and listeriosis cases. The findings in this study improve our understanding of the association of biofilm production with phylogenetic lineage and serotype in L. monocytogenes.

## INTRODUCTION

Listeria monocytogenes is a major bacterial cause of foodborne deaths, exhibiting the highest case fatality rate among foodborne pathogens in the United States and the European Union (1, 2). Serious clinical symptoms manifested by L. monocytogenes include sepsis, meningitis, and encephalitis, particularly in newborn and unborn babies, elderly people, immunocompromised individuals, and pregnant females (3–5). L. monocytogenes can be grouped into four genetic lineages and 13 serotypes (6). Among the four genetic lineages, lineages I and II harboring serotypes 1/2b and 4b (lineage I) and 1/2a (lineage II) account for over 95% of human listeriosis cases (3, 7, 8). Serotypes 1/2b and 4b strains are overrepresented among human isolates from listeriosis outbreaks compared to serotype 1/2a strains, which are commonly isolated from foods, natural environments, and sporadic listeriosis cases (6). Multilocus sequence typing (MLST) based on the sequences of 7 housekeeping genes is broadly used to investigate the phylogenetic structure of L. monocytogenes (9). The clonal complex (CC) system determined by the 7-gene MLST scheme is closely related to the serotype and genetic lineages of L. monocytogenes (4, 10, 11). Additionally, studies show that MLST CCs are congruent with the cluster groupings of core genome multilocus sequence typing (cgMLST) (4, 12).

L. monocytogenes is of great concern to public health and the food industry because of its frequent implication in deadly outbreaks and costly food recalls (13). Various kinds of foods are vulnerable to L. monocytogenes contamination, including dairy products, soft cheese, refrigerated smoked seafood, ready-to-eat foods, sprouts, and cantaloupe melons (14). Due to the serious health consequences of listeriosis, strict food regulations regarding L. monocytogenes contamination have been established in many countries. Particularly, the United States adopts a zero-tolerance policy on L. monocytogenes for ready-to-eat foods (15). However, it is extremely difficult to prevent food contamination by L. monocytogenes because this pathogenic bacterium is ubiquitous in the environment surrounding food production and processing and is capable of developing biofilms on food-processing facilities (16–18). Biofilms are highly tolerant to disinfectants and serve as a persistent reservoir for cross-contamination of foods (18–20). L. monocytogenes can cause persistent contamination of food-processing environments (21–23), which increases the risks of cross-contamination of finished products and can lead to outbreaks (23). For instance, the same clone of L. monocytogenes from a single processing plant caused sporadic listeriosis in 1988 and a multistate outbreak in the United States in 2000, suggesting that the L. monocytogenes strain persisted in the food-processing facilities for at least 12 years (23).

Phylogenetic lineages and serotypes are closely related to human infection and food contamination by L. monocytogenes. Serotypes 1/2a, 1/2b, and 4b account for the majority of human listeriosis cases and food contamination (3, 7, 8). Biofilm production in L. monocytogenes is related to persistent food contamination and thereby human infection (24, 25). However, studies have presented conflicting results regarding the association between biofilm formation and phylogeny and serotype (5, 26). Here, we measured biofilm production in L. monocytogenes using 98 clinical isolates collected by the Minnesota Department of Health from outbreaks and sporadic cases and evaluated the association of biofilm formation with phylogeny, serotypes, and genotypes. We discovered that the ability to form biofilms is related to serotype and phylogeny, and the modification of teichoic acids with sugars plays a critical role in strong biofilm production in serotypes 1/2a and 1/2b, which are frequently involved in food contamination and human infection.

## RESULTS

### Phylogenetic association of biofilm production in L. monocytogenes.

**FIG 1:**
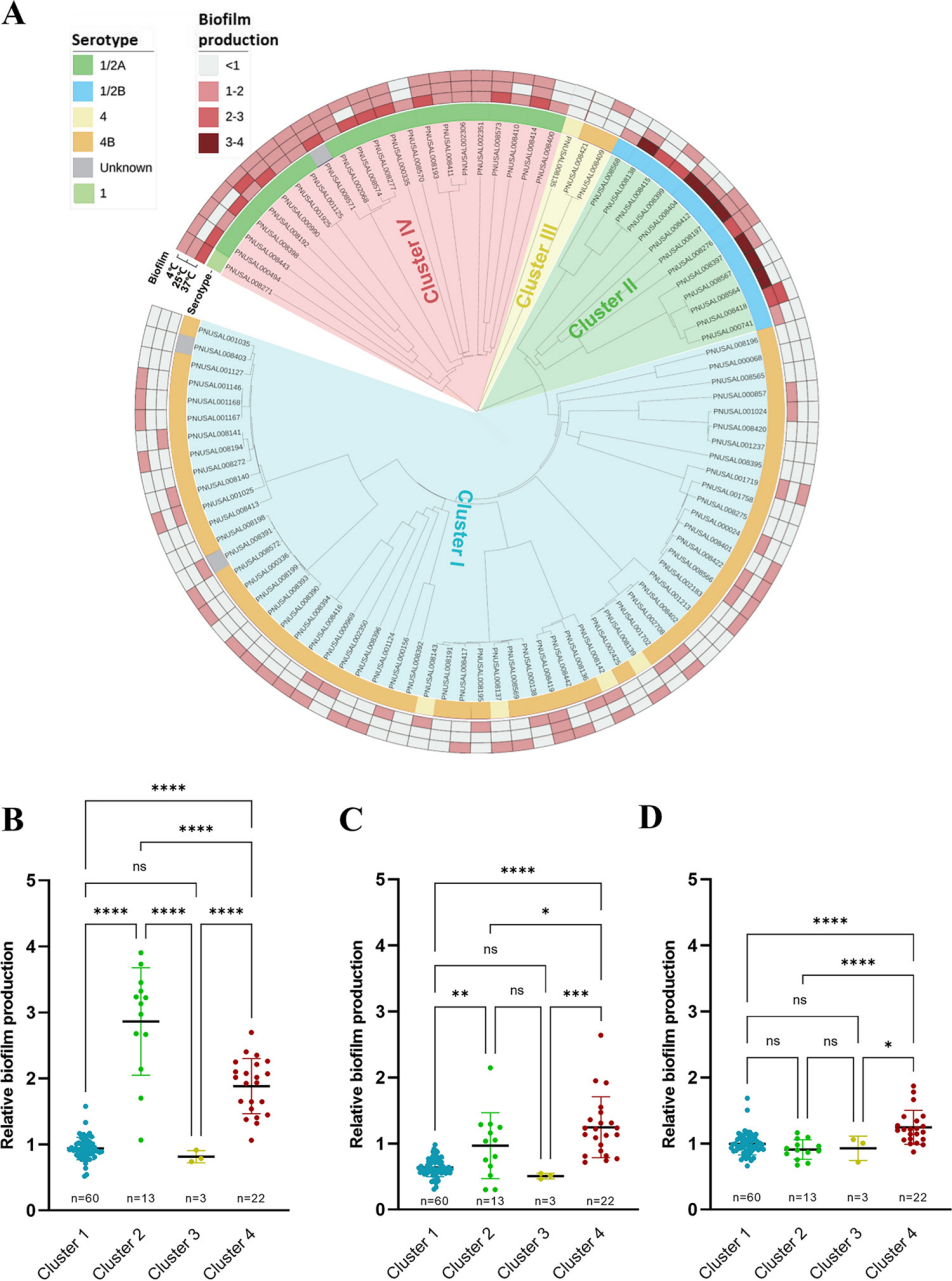
Phylogenetic association of 98 clinical isolates of L. monocytogenes with biofilm productivity. (A) A phylogenetic tree was generated using the cgMLST scheme based on 1,748 loci (27). The relative level of biofilm production was measured by comparing biofilm levels between the clinical isolates and a control strain (L. monocytogenes ATCC 19115). (B to D) The biofilm productivity of the four phylogenetic clusters at 37°C (B), 25°C (C), and 4°C (D). Statistical analysis was conducted by the one-way analysis of variance (ANOVA) with Tukey’s multiple-comparison tests; ns, nonsignificant; *, *P < *0.05; **, *P < *0.01; ***, *P < *0.001; ****, *P < *0.0001.

We first evaluated biofilm production in 98 serotyped and whole-genome-sequenced clinical strains of L. monocytogenes isolated by the Minnesota Department of Health from listeriosis outbreaks and sporadic cases from 2004 to 2017 (Table S2 in the supplemental material). A phylogenetic analysis was conducted using the cgMLST scheme based on 1,748 loci in L. monocytogenes genomes (27). cgMLST analysis grouped these strains into four phylogenic clusters, which were closely related to serotypes (Fig. 1A). Clusters 1 and 3 consisted of serotype 4 and 4b strains except two nontypeable strains in cluster 1, while clusters 2 and 4 were composed of primarily serotype 1/2b and 1/2a strains, respectively, except one nontypeable strain and one serotype 1 strain in cluster 4 (Fig. 1A).

The level of biofilm production in the 98 strains was measured at three different temperatures, including 37°C (the optimal growth temperature for L. monocytogenes and the body temperatures of humans), 25°C (room temperature), and 4°C (a refrigeration temperature). Considering batch-to-batch variations, we included a control strain (L. monocytogenes ATCC 19115) in each plate of the biofilm assay to assess the relative level of biofilm production in comparison with that of the control strain. L. monocytogenes ATCC 19115 belongs to serotype 4b and is used as a quality control strain for bacterial identification (28). The clinical strains of L. monocytogenes exhibited a wide range of variation in levels of biofilm production, which is consistent with previous studies categorizing L. monocytogenes strains as weak, moderate, and strong biofilm producers based on biofilm-forming ability (29). Remarkably, the level of biofilm production was associated with phylogenetic clusters and serotypes (Fig. 1A). Whereas the isolates in clusters 1 and 3 formed biofilms at low levels, strong biofilm-forming strains belonged to clusters 2 and 4 (Fig. 1). Particularly, cluster 2 (serotype 1/2b) strains exhibited strong biofilm production at 37°C (Fig. 1B), and the levels of biofilm production of cluster 4 (serotype 1/2a) strains were significantly elevated at all tested temperatures (Fig. 1B to D).

### Association of MLST CC with biofilm production in L. monocytogenes.

**FIG 2:**
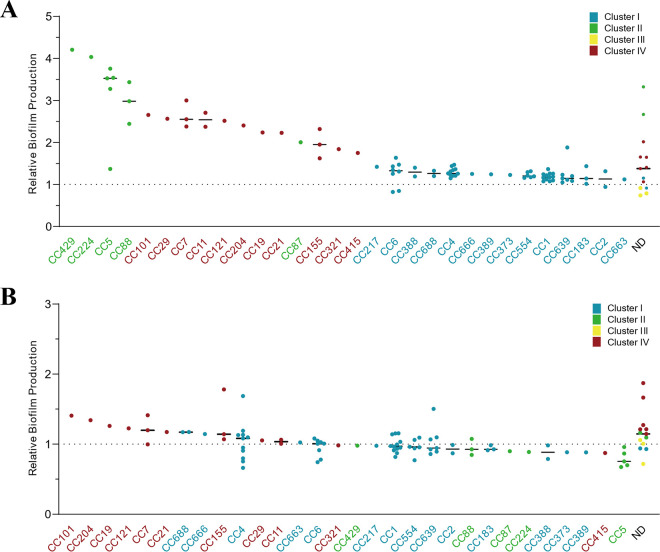
(A and B) Biofilm production of the CCs of 98 clinical isolates of L. monocytogenes at 37°C (A) and 4°C (B). The four phylogenetic clusters identified by cgMLST (Fig. 1A) are indicated in different colors. A solid black line indicates the mean; UT, untypeable.

The MLST CCs of the strains belonging to cluster 2, including CC429, CC224, CC5, and CC88, were major clones of strong biofilm producers at 37°C, followed by cluster 4 CCs (Fig. 2A). The strains of CC5 and CC88 in cluster 2 and CC7 and CC11 in cluster 4 formed biofilms at higher levels than those of the CCs in cluster 1 with statistical significance (Table S1). At 4°C, the strains of cluster 4 CCs, such as CC7 and CC155, were strong biofilm producers (Fig. 2B); however, the differences were not statistically significant. CC7 is a clone highly prevalent in dairy farm and animal clinical cases in the United States (30, 31) and is also common in human listeriosis cases (30, 32). CC155 strains have been isolated from humans, foods, and food-processing environments (33, 34). CC121 is a clone related to persistent contamination of food production environments and is dominant in food isolates (4, 7). Although there was only one CC121 isolate among the tested strains, it exhibited a strong biofilm-forming ability at 4°C (Fig. 2B). Pathogenic potential of L. monocytogenes is associated with MLST CCs (4, 35). Hypervirulent CCs (e.g., CC1, CC2, CC4, and CC6) frequently involved in outbreaks belong to serotype 4b (4, 7). Similarly, the dominant clones of the clinical isolates used in the study were CC1 (12.2%), CC4 (11.2%), and CC6 (9.2%), all of which belong to cluster 1 (serotype 4b) (Fig. 2A). These hypervirulent clones were overall all weak biofilm producers at 37°C (Fig. 2A), whereas CC4 exhibited strong biofilm-forming ability at 4°C (Fig. 2B). These results show that genotypes are related to biofilm production in association with temperatures.

### Identification of genes unique to strong biofilm producers.

**FIG 3:**
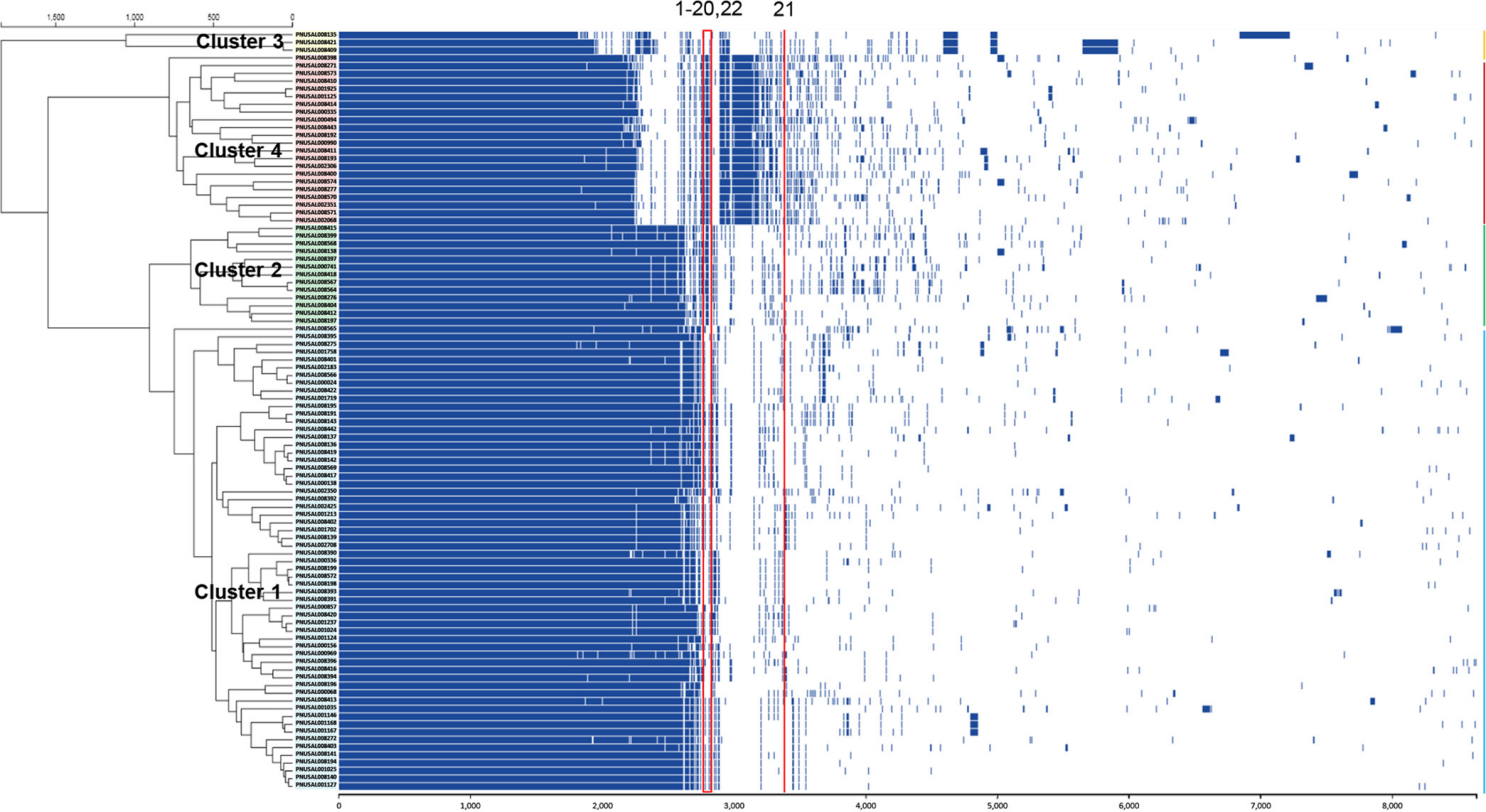
Linearized pan-genomic view of 98 L. monocytogenes strains. The assembled genomes of 98 L. monocytogenes strains were annotated with Prokka v1.14.6 and used for a pan-genome analysis using Roary v3.11.2. The resulting presence and absence matrix of orthologous genes was visualized using FriPan. The red box and line indicate the location of the genes unique to strong biofilm formers listed in Table 1. The gene numbers are indicated on top.

A pan-genome analysis also grouped the 98 strains into the same four clusters of cgMLST (Fig. 3). To identify genes uniquely present in L. monocytogenes strains forming biofilms at high levels, a pan-genome analysis was conducted by comparing the genomes of strong biofilm producers (the strains indicated with dark red in biofilm levels in Fig. 1A) and those of weak biofilm formers (those indicated with white in Fig. 1A) at 37°C. We compared biofilm levels at 37°C because at this temperature, the ability to form biofilms could be easily differentiated (Fig. 1A and B). The analysis identified 22 genes (Table 1), most of which were related to the synthesis or modification of teichoic acids. Teichoic acids constitute 60% of the total dry mass of the cell wall of L. monocytogenes and are the major soluble carbohydrates in the extracellular matrix of *Listeria* biofilms (36, 37). Teichoic acids are either anchored to membrane lipids (lipoteichoic acid; LTA) or associated with the peptidoglycan layer (wall teichoic acid; WTA) (38). WTAs in L. monocytogenes are major antigenic determinants and mediate antibiotic resistance (39), virulence (40), and phage susceptibility (41). WTAs are composed of ribitol-phosphate subunits, whose hydroxyl groups can be substituted by diverse monosaccharides (42). The identified genes included the *tag* (teichoic acid glycerol) genes for the synthesis of LTAs and *tar* (teichoic acid ribitol) genes mediating the synthesis of WTAs (38, 43), whose homologs were available in cluster 1 strains (Table 1). Whereas *N*-acetylglucosamine (GlcNAc) is commonly present in WTAs of serotypes 1/2 and 4b, l-rhamnose decorates WTAs in serogroup 1/2, and d-glucose and d-galactose modify WTAs in serotype 4b (42). The *rml* genes are responsible for the synthesis of dTDP-l-rhamnose and the incorporation of l-rhamnose to WTAs in serogroup 1/2 (39, 44). The *ami* gene encodes an autolysin amidase noncovalently associated with the cell wall and is related to biofilm formation in L. monocytogenes (45, 46). The *inlB* gene encodes internalin B, a surface-bound protein involved in the listerial invasion of cells (47). The *rml* operon genes, *inlB*, *ami* (*lmo2558*), and a few other genes with unknown functions, were present in strong biofilm producers and were absent from cluster 1 consisting of weak biofilm producers (Table 1).

**TABLE 1:** Genes uniquely present in strong biofilm producers of L. monocytogenes

No.	Gene	Annotation	EGD-e^*c*^	Availability in cluster 1^*a*^
1	*tarL*	Teichoic acid poly-(ribitol-phosphate) polymerase	*lmo1085*	*tarL*, different gene^*b*^
2	*tarI*	Ribitol-5-phosphate cytidylyltransferase	*lmo1086*	*tarI*, partially similar
3	*tarJ*	Ribulose-5-phosphate reductase 1	*lmo1087*	*tarJ*, partially similar
4	*tagG*	Teichoic acid translocation permease protein	*lmo1074*	*tagG*, partially similar
5	*tagB*	Teichoic acid glycerol-phosphate primase	*lmo1088*	*tagB*, partially similar
6	*tagD*	Glycerol-3-phosphate cytidylyltransferase	*lmo1089*	*tagD*, partially similar
7	*galU*	UTP-glucose-1-phosphate uridylyltransferase	*lmo1078*	*gtaB*, partially similar
8	*inlB*	Internalin B	*lmo0434*	*intB*, partially similar
9	*gtcA*	Cell wall teichoic acid glycosylation protein	*lmo2549*	*yfdG*, partially similar
10	*murZ*	UDP-*N*-acetylglucosamine 1-carboxyvinyltransferase 2	*lmo2552*	*murAB*, partially similar
11	*ami*	Autolysin	*lmo2558*	Absent
12	*rmlT*	Putative glycosyltransferase	*lmo1080*	Absent
13	*rmlA*	Glucose-1-phosphate thymidylyltransferase 1	*lmo1081*	Absent
14	*rmlC*	dTDP-4-dehydrorhamnose 3,5-epimerase	*lmo1082*	Absent
15	*rmlB*	dTDP-glucose 4,6-dehydratase 2	*lmo1083*	Absent
16	*rmlD*	dTDP-4-dehydrorhamnose reductase	*lmo1084*	Absent
17	Unknown	Putative glycosyltransferase	*lmo2550*	Absent
18	Unknown	Hypothetical protein	*lmo1079*	Absent
19	Unknown	Hypothetical protein	*lmo1188*	Absent
20	Unknown	Hypothetical protein	*lmo1068*	Partially similar
21	Unknown	Hypothetical protein	*lmo0126*	Absent
22	Unknown	Hypothetical protein	*lmo0127*	Partially similar

a The analysis was conducted in comparison with PNUSAL001146.

b The translated amino acid sequence similarity is 29.49%.

c The homologous gene in L. monocytogenes EGD-e, a serotype 1/2a strain (GenBank accession number: NC_003210.1)

### Association of strong biofilm production with the identified genes in L. monocytogenes.

**FIG 4:**
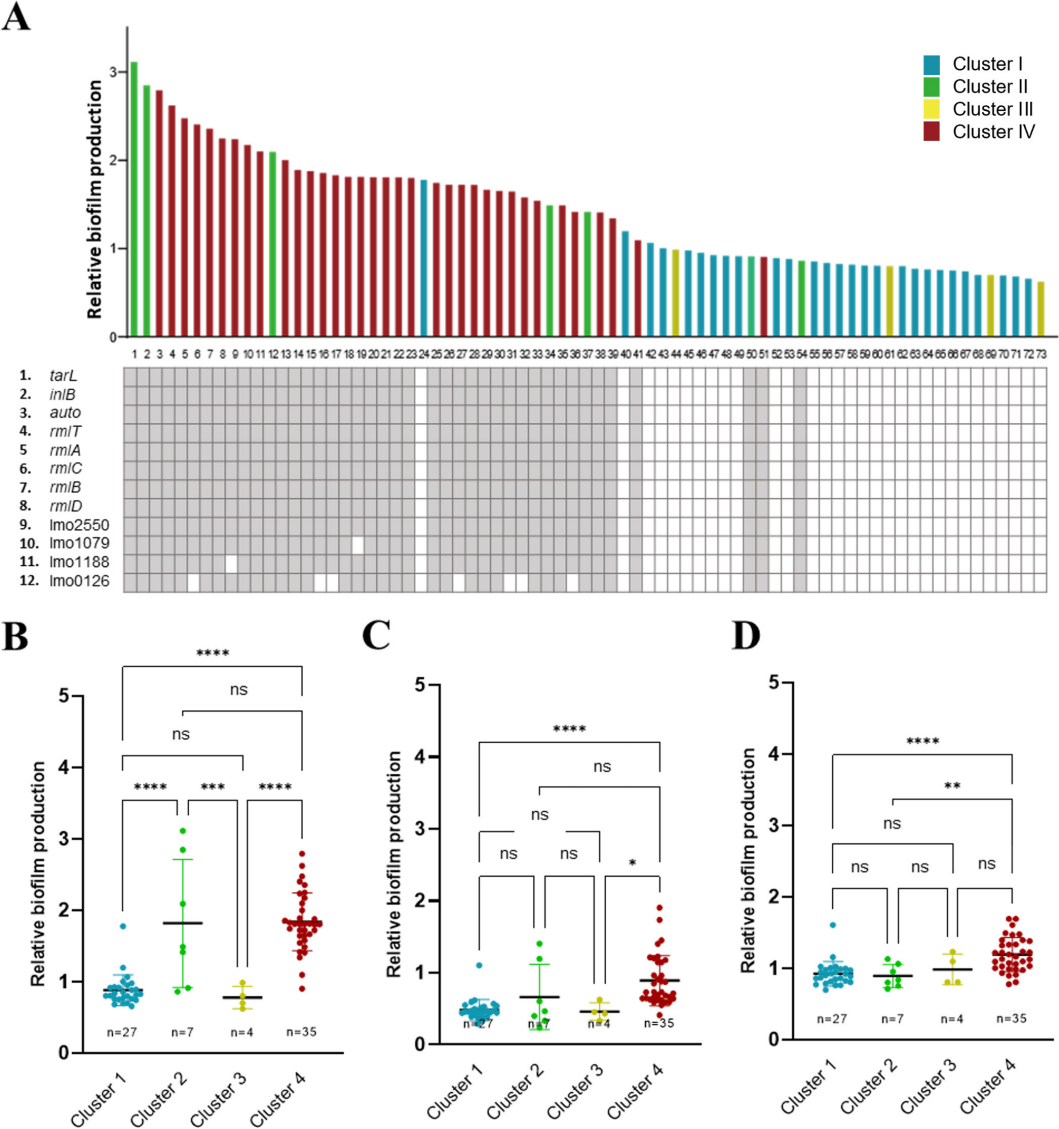
Relative biofilm productivity of additional 73 L. monocytogenes strains. (A) Biofilm production of L. monocytogenes in association with the presence and absence of genes identified by the pan-genome analysis (Table 1). The relative level of biofilm production was measured by comparing biofilm levels between the isolates and a control strain (L. monocytogenes ATCC 19115). The filled and open squares represent the presence and absence of a gene, respectively. The numbers of the isolates beneath the figure correspond to those in Table S2 in the supplemental material. (B to D) The biofilm productivity of the four phylogenetic clusters at 37°C (B), 25°C (C), and 4°C (D). A solid black line indicates the mean. Statistical analysis was conducted with the one-way ANOVA with Tukey’s multiple-comparison tests; ns, nonsignificant; *, *P < *0.05; **, *P < *0.01; ***, *P < *0.001; ****, *P < *0.0001.

In order to confirm whether the 22 genes identified by the pan-genome analysis are associated with strong biofilm production (Table 1), we decided to validate the association by testing biofilm formation in an additional 73 strains of L. monocytogenes, which were whole-genome-sequenced isolates from clinical cases, foods, and environmental sources (Table S3). As a blind test, we first measured the levels of biofilm production in the 73 strains without knowing their phylogenetic information and correlated the presence of the identified genes to the phylogenetic clusters determined by cgMLST analysis. For this, we targeted the genes whose presence and absence are clearly differentiated depending on the phylogenetic cluster. Consistent with results from the original 98 strains (Fig. 1), cluster 2 and 4 strains exhibited a strong biofilm-forming activity (green and red bars in Fig. 4A) compared to cluster 1 strains (blue bars in Fig. 4A). Cluster 1 and 3 strains were weak biofilm producers, and cluster 2 showed the highest median value at 37°C despite a wide range of variations, and the strains in cluster 4 exhibited overall high levels of biofilm formation at the three tested temperatures (Fig. 4B to D), exhibiting the same patterns observed in the first batch of 98 clinical strains (Fig. 1B to D). These results confirm that the ability of strong biofilm formation is phylogenetically related in L. monocytogenes.

### Sugar modification of WTAs with l-rhamnose mediates strong biofilm production in L. monocytogenes.

**FIG 5:**
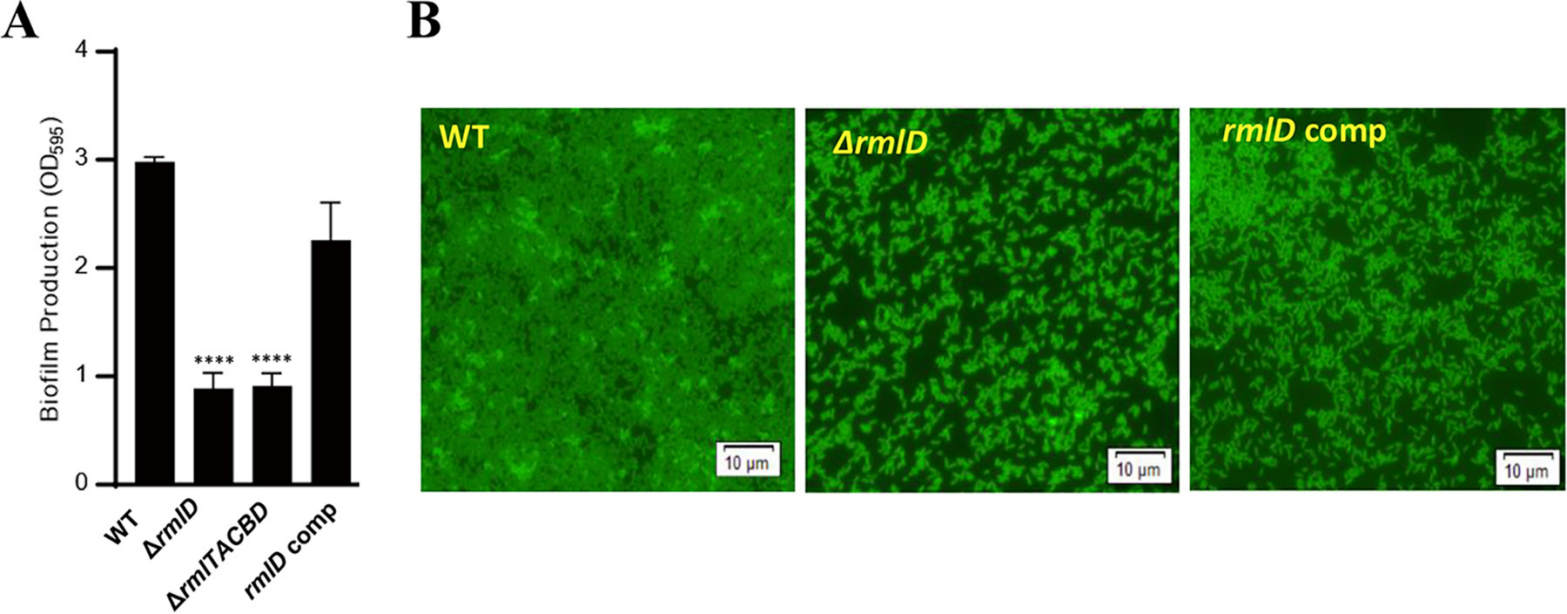
Effects of l-rhamnosylation on biofilm formation in L. monocytogenes. (A) Defective biofilm production in Δ*rmlD* and Δ*rmlTACBD* mutants. Statistical analysis was conducted with the Student’s *t* test in comparison with wild-type (WT); ****, *P < *0.0001; *rmlD* comp, a *rmlD*-complemented strain. The results are representative of three independent experiments, which produced similar results. (B) Compromised biofilm maturation in a Δ*rmlD* mutant. Fluorescence microscopic images show that a Δ*rmlD* mutant cannot produce mature biofilms compared to WT.

The *rml* operon genes involved in the modification of WTAs with l-rhamnose were consistently related to strong biofilm producers throughout the experiments using 98 strains (Table 1) and an additional 73 strains (Fig. 4A). Rhamnosylation of WTAs in serogroup 1/2 is mediated by the *rmlACBD* locus and *rmlT*, which encodes a rhamnosyltransferase (41). In order to evaluate the association of the *rml* genes with biofilm production, we constructed in-frame deletion mutants of *rmlD*, which encodes dTDP-4-dehydrorhamnose reductase mediating the final step of dTDP-l-rhamnose synthesis (48) and the entire operon (*rmlTACBD*). For the mutagenesis, we used PNUSAL008411, a serotype 1/2a strain in cluster 4, which was isolated from a sporadic listeriosis case and produced biofilms at high levels at all tested temperatures (Fig. 1A). Notably, knockout mutations of *rmlD* and *rmlTACBD* significantly reduced the levels of biofilm formation (Fig. 5A). Microscopic analysis revealed that mutants defective in l-rhamnosylation could adhere to a surface and formed microcolonies but could not develop mature biofilm structures (Fig. 5B). These results demonstrate that l-rhamnosylation of WTAs, which is specific to serogroup 1/2, is involved in strong biofilm production in L. monocytogenes.

## DISCUSSION

Our results first demonstrate that strong biofilm production in serotypes 1/2a and 1/2b results from the modification of WTAs with l-rhamnose. Serotypes 1/2a, 1/2b, and 4b are most frequently implicated in food contamination and listeriosis cases (3, 7, 8). Serotypes 1/2b and 4b are often involved in listeriosis outbreaks (6), whereas serotype 1/2a is overrepresented in isolates from foods and food-related environments (6). Studies to date have shown contradictory results regarding the relationship between biofilm-forming ability and serotypes and phylogenetic lineages in L. monocytogenes (5, 49). However, the majority of studies show that serotype 1/2 isolates are generally strong biofilm producers compared with serotype 4b isolates (50–53). Additionally, L. monocytogenes strains isolated from foods, primarily serotype 1/2a isolates, have higher biofilm-forming capabilities than clinical isolates (4). Consistent with these reports, our data demonstrate that serotype 1/2 strains exhibit significantly higher levels of biofilm production than serotype 4b strains (Fig. 1). Previous studies measuring biofilm production in L. monocytogenes have presented optical density (OD) values from biofilm assays performed with crystal violet staining, which normally generates wide batch-to-batch variations and makes it difficult to compare biofilm levels when plenty of strains are tested. To address this technical issue, we included a control strain in every biofilm assay plate and determined the relative level of biofilm production in comparison with that of the control strain. This approach allowed for comparison of the levels of biofilm production in 98 strains of L. monocytogenes and minimized batch-to-batch variations in data analysis.

Studies have shown that the level of biofilm production in L. monocytogenes is higher at 37°C than at lower temperatures (49, 54). However, it is not known whether serotype can influence temperature-dependent biofilm production in L. monocytogenes. Notably, our data demonstrate that the effect of temperature on biofilm production is related to the serotype of L. monocytogenes (Fig. 1). The strong biofilm-forming activity in serogroup 1/2 can affect food contamination and human infection in association with temperature (Fig. 1B to D). The deadly cantaloupe outbreak in the United States in 2011 was caused by 1/2a and 1/2b strains (55). Based on our results, it may be because 1/2a and 1/2b strains are strong biofilm producers at 25°C and 37°C (Fig. 1B and C) and can form biofilms on cantaloupe surfaces at warm temperatures in farming environments (56). Additionally, the strong biofilm-forming activity of serotype 1/2a at low temperatures may also contribute to the contamination of dairy and ready-to-eat foods processed and stored at low temperatures. Whereas serotype 1/2a strains (cluster 4) show strong biofilm-forming capabilities over various temperature ranges, serotype 1/2b strains (cluster 2) are the strongest biofilm producers at 37°C (Fig. 1). Considering that 37°C is the average normal body temperature of humans, we can speculate that the strong biofilm-forming activity of serotype 1/2b at 37°C may contribute to human infection with L. monocytogenes. Interestingly, two serotype 1/2b strains (PNUSAL008564 and PNUSAL008567) isolated from listeriosis patients with febrile gastroenteritis exhibited very strong biofilm production (Fig. 1). Strong biofilm formation activity at 37°C can possibly facilitate the adhesion of these 1/2b strains to epithelial cells in the course of developing gastroenteritis. However, future studies are required to validate this hypothesis.

Remarkably, our results first demonstrate that sugar decoration of WTAs with l-rhamnose is responsible for strong biofilm production in serotype 1/2a and 1/2b (Fig. 5). The association of teichoic acids with biofilm formation has been reported (46). The absence of GlcNAc, a common sugar in WTAs of serotypes 1/2 and 4b, in L. monocytogenes leads to modification of biofilm structures and tolerance to rinsing and cleaning procedures (57). Treatment with subinhibitory concentrations of tunicamycin, a WTA-biosynthesis-inhibiting antibiotic, reduces biofilm formation in L. monocytogenes (58). Using single nucleotide polymorphism (SNP) analysis in comparison with reference strains, Hsu et al. discovered *rmlA*, encoding the first enzyme for dTDP-l-rhamnose biosynthesis, which can be associated with biofilm formation in L. monocytogenes (59). Notably, our data show that rhamnosylation is required for strong biofilm production in serotype 1/2 by affecting biofilm maturation in L. monocytogenes (Fig. 5). Moreover, l-rhamnosylation of WTAs is necessary for retaining the Ami autolysin in L. monocytogenes, and its autolytic activity is decreased in the absence of l-rhamnosylated WTAs (60). Since extracellular DNA (eDNA) is an important component of biofilm matrices and the autolysis-mediated release of eDNA by autolysins can influence biofilm production (61), rhamnosylation of WTAs can influence biofilm formation by reducing autolysis-mediated eDNA release. However, a knockout mutation of Δ*ami* only resulted in a minor (~20%) reduction in biofilm production (Fig. S1 in the supplemental material), suggesting that the effect of rhamnosylation on biofilm production through the function of autolysins is not primary. Presumably, rhamnosylation can facilitate biofilm formation by altering the physicochemical features of WTAs, anionic polymers composed of alternating phosphate and ribitol (42). The modification of WTAs with l-rhamnose delays the penetration of the cell wall by antimicrobial peptides and whereby affects their contact with the membrane of L. monocytogenes, increasing antimicrobial resistance (39). This indicates that rhamnosylation alters the integrity of cell walls in L. monocytogenes. l-Rhamnose is chemically unique compared to other hexoses. Rhamnose is a deoxy sugar lacking a hydroxyl group and has five oxygen molecules, while most other hexoses, such as glucose and galactose, have six. Whereas most naturally occurring sugars in nature are in d-form, the predominant natural form of rhamnose is l-form (62, 63). Compared to the WTAs modified with d-glucose and d-galactose in serotype 4b (42), the modification of WTAs with l-rhamnose in serotype 1/2 may alter the physicochemical features of WTAs, the major extracellular polysaccharides in *Listeria* biofilms (36, 37).

In summary, our results demonstrate that biofilm production in L. monocytogenes is associated with phylogeny and serotype and that l-rhamnosylation of WTAs is responsible for strong biofilm production in serotype 1/2, which is frequently involved in food contamination and human infections. Based on the sugar modification of WTAs in L. monocytogenes, the removal of l-rhamnose serologically converts serotype 1/2 to serotype 3, which has WTAs decorated with only GlcNAc (41). Serotypes 1/2a and 1/2b were collectively responsible for 30% of *Listeria* infections in the United States from 1996 to 2020, and serotype 4b caused 28%. However, all the rest of the serotypes, including 3a, 3b, and 3c, only accounted for 4% in the same time frame (https://wwwn.cdc.gov/foodnetfast/). Besides genetic differences in these serotypes, it will be important future research to evaluate the effects of l-rhamnosylation of WTAs on food contamination and human infection by facilitating biofilm formation in L. monocytogenes.

## MATERIALS AND METHODS

### Bacterial strains and culture.

L. monocytogenes ATCC 19115 was purchased from ATCC, and 171 strains of L. monocytogenes were isolated and whole-genome sequenced by the Minnesota Department of Health from 2004 to 2017. L. monocytogenes strains were aerobically cultured at 37°C on brain heart infusion (BHI) medium.

### Biofilm assay.

Biofilm assays were conducted as described previously (25) with some modifications. Briefly, bacterial suspension was prepared from overnight cultures of L. monocytogenes, diluted with fresh BHI medium to an optical density at 600 nm (OD_600_) of 0.1, and placed into a 24-well plate. After 24 h for biofilm production at 37°C and 25°C or 72 h at 4°C, biofilms were washed twice with 1 mL of phosphate-buffered saline (PBS; pH 7.4). Plates were completely dried in a drying oven at 60°C for 30 min, and 250 μL of 1% crystal violet was administered to each well. After incubation at room temperature for 40 min, wells were washed with 1 mL of PBS three times. Plates were dried at room temperature for 3 h, and the remaining crystal violet was eluted with 500 μL of elution buffer (10% acetic acid and 30% methanol). The OD_595_ was detected by a plate reader (Varioskan, Thermo Fisher). The experiments were repeated three times.

### Fluorescence microscopic analysis of biofilms.

Biofilm formation was also analyzed by fluorescence microscopy. Biofilms were developed on a circle cover glass in a 24-well plate for 24 h at 37°C. Biofilm samples were washed twice with PBS and fixed with 4% paraformaldehyde for 30 min at room temperature. The biofilms were then washed with PBS and stained with SYTO9 (Thermo Fisher). After washing, biofilms were analyzed with a fluorescence microscope (Nikon, Japan).

### Construction of in-frame deletion mutants of *rmlD* and *rmlTACBD* and an *rmlD*-complemented strain.

The deletion mutants of *rmlD* and *rmlTACBD* were generated using the pHoss1 plasmid as previously described (64). The upstream and downstream flanking regions of the genes were amplified by PCR using A/B and C/D primers (Table S4 in the supplemental material). The primers used for this study are listed in Table S3. The PCR fragments were assembled by overlap extension PCR using the two flanking primers (A and D). The SalI- and NcoI-digested PCR amplicons were inserted into the pHoss1 plasmid to create suicide vectors by transforming into Escherichia coli DH5α. The suicide plasmids were introduced to L. monocytogenes PNUSAL008411 by electroporation. Allelic exchange in the mutants was confirmed by PCR. An *rmlD*-complemented strain was constructed using pL2 (65). A DNA fragment containing the intact copy of *rmlD* was amplified with Clon-*rmlD*-SalI-F and Clon-*rmlD*-NotI-R and cloned into pL2 digested with SalI and NcoI. The constructed plasmid was introduced to a Δ*rmlD* mutant by electroporation followed by selective growth on BHI agar plates supplemented with 25 μg/mL chloramphenicol.

### *Listeria* cgMLST analysis.

cgMLST was performed in BioNumerics version 7.6 (bioMérieux, France) using a scheme containing 1,748 loci (27). The whole-genome sequencing (WGS) plug-in tools in BioNumerics provide assembly-free and assembly-based calling to identify alleles. Briefly, *de novo* assembly was performed using SPAdes (version 3.7.1) with the parameters of 5× minimum coverage, 20× expected coverage, 500 minimum contig length, and 10% low coverage filtering threshold. After *de novo* assembly, the sequences obtained were scanned with the assembly-based call. The minimum homology for allele calling and minimum similarity to call new alleles for assembly-based call were 85% and 70%, respectively. The kmer size (35 bp), minimum coverage (3×), minimum forward coverage (1×), and minimum reverse coverage (1×) were set for assembly free call. Consequently, combined alleles from assembly-based and assembly-free calls were included in this analysis after removing the discrepant results between the two algorithms. Clustering was analyzed using the categorical difference coefficient, and the newick file created by the unweighted pair group method with arithmetic mean (UPGMA) algorithm was exported from BioNumerics. The phylogenetic tree was visualized with the ITOL interactive website (https://itol.embl.de/upload.cgi).

### Pan-genome analysis.

The assembled genome files of 98 L. monocytogenes strains were downloaded from NCBI, and all genome sequences were annotated using Prokka v1.14.6 with default parameters (66). The output files of Prokka were used to perform pan-genome analysis of L. monocytogenes using Roary v3.11.2 (67), followed by visualization of results via Fripan (http://drpowell.github.io/FriPan/).
