# Supplementary material for: Sugar Modification of Wall Teichoic Acids Determines Serotype-Dependent Strong Biofilm Production in Listeria monocytogenes
Source: Microbiol Spectr. 2022 Oct 3;10(5):e02769-22. doi: 10.1128/spectrum.02769-22 (PMC9603678; doi:10.1128/spectrum.02769-22)
Supplement: Supplemental file 1 — Supplemental material. Download spectrum.02769-22-s0001.pdf, PDF file, 0.3 MB [file spectrum.02769-22-s0001.pdf]

SUPPLEMENTAL DATA

Table S1. Statitical analysis of differences in biofilm production between CCs†.

	Significance††	Mean Diff.	95% CI of diff.
CC5 vs. CC6	****	1.829	0.9305 to 2.727
CC5 vs. CC388	***	1.798	0.4798 to 3.116
CC5 vs. CC688	***	1.828	0.5103 to 3.147
CC5 vs. CC4	****	1.798	0.9486 to 2.648
CC5 vs. CC554	****	1.867	0.9134 to 2.821
CC5 vs. CC1	****	1.906	1.068 to 2.745
CC5 vs. CC639	****	1.849	0.9268 to 2.772
CC5 vs. CC183	****	1.895	0.7449 to 3.046
CC5 vs. CC2	***	1.963	0.6448 to 3.281
CC5 vs. UT	****	1.277	0.4484 to 2.106
CC88 vs. CC6	****	1.69	0.6230 to 2.756
CC88 vs. CC388	*	1.659	0.2207 to 3.097
CC88 vs. CC688	**	1.689	0.2512 to 3.127
CC88 vs. CC4	****	1.659	0.6331 to 2.685
CC88 vs. CC554	****	1.728	0.6143 to 2.842
CC88 vs. CC1	****	1.767	0.7502 to 2.784
CC88 vs. CC639	****	1.71	0.6230 to 2.797
CC88 vs. CC183	***	1.756	0.4700 to 3.043
CC88 vs. CC2	**	1.824	0.3857 to 3.262
CC88 vs. UT	*	1.138	0.1293 to 2.147
CC7 vs. CC6	**	1.38	0.3137 to 2.447
CC7 vs. CC4	**	1.35	0.3238 to 2.376
CC7 vs. CC554	**	1.419	0.3050 to 2.533
CC7 vs. CC1	***	1.458	0.4409 to 2.475
CC7 vs. CC639	**	1.401	0.3137 to 2.488
CC7 vs. CC183	*	1.447	0.1607 to 2.733
CC7 vs. CC2	*	1.515	0.07633 to 2.953
CC11 vs. CC6	*	1.276	0.03076 to 2.522
CC11 vs. CC4	*	1.246	0.03487 to 2.457
CC11 vs. CC554	*	1.315	0.02867 to 2.601
CC11 vs. CC1	*	1.354	0.1506 to 2.557
CC11 vs. CC639	*	1.297	0.03370 to 2.560

† The analysis was conducted with one-way analysis of variance (ANOVA) followd by Tukey's multiple comparisons test.
†† *: P<0.05, **:P<0.01, ***:P<0.001, and ****: P<0.0001.

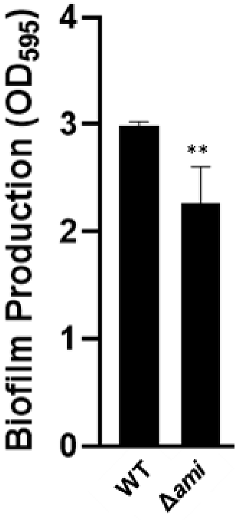

Fig. S1. Biofilm reduction by a knockout mutation of *ami* (*lmo2558*) encoding an autolysin. Statistical analysis was conducted with the Student's *t*-test in comparison with WT. **: $P<0.01$. The results are representative of three independent experiments, which produced similar results.

Table S2. The accession numbers of 98 *L. monocytogenes* clinical strains used in the study

Strain	Accession no.	Strain	Accession no.
PNUSAL008135	SAMN16355294	PNUSAL008191	SAMN16392407
PNUSAL008421	SAMN17098147	PNUSAL008143	SAMN16355296
PNUSAL008409	SAMN16583326	PNUSAL008442	SAMN17098082
PNUSAL008398	SAMN16569099	PNUSAL008137	SAMN16355295
PNUSAL008271	SAMN16439277	PNUSAL008136	SAMN16355297
PNUSAL008573	SAMN16689239	PNUSAL008419	SAMN16583331
PNUSAL008410	SAMN16583333	PNUSAL008142	SAMN16355293
PNUSAL001925	SAMN04335498	PNUSAL008569	SAMN16689232
PNUSAL001125	SAMN03153524	PNUSAL008417	SAMN16583329
PNUSAL008414	SAMN16583315	PNUSAL000138	SAMN02351392
PNUSAL000335	SAMN02413353	PNUSAL002350	SAMN05504542
PNUSAL000494	SAMN02566966	PNUSAL008392	SAMN16569136
PNUSAL008443	SAMN17098139	PNUSAL002425	SAMN05604758
PNUSAL008192	SAMN16392404	PNUSAL001213	SAMN03255936
PNUSAL000990	SAMN03067799	PNUSAL008402	SAMN16569141
PNUSAL008411	SAMN16583328	PNUSAL001702	SAMN04018933
PNUSAL008193	SAMN16392413	PNUSAL008139	SAMN16355292
PNUSAL002306	SAMN05372196	PNUSAL002708	SAMN06111293
PNUSAL008400	SAMN16569101	PNUSAL008390	SAMN16569134
PNUSAL008574	SAMN16689237	PNUSAL000336	SAMN02413354
PNUSAL008277	SAMN16439298	PNUSAL008199	SAMN16392408
PNUSAL008570	SAMN16689235	PNUSAL008572	SAMN16689236
PNUSAL002351	SAMN05504540	PNUSAL008198	SAMN16392410
PNUSAL008571	SAMN16689238	PNUSAL008393	SAMN16569140
PNUSAL002068	SAMN04543918	PNUSAL008391	SAMN16569138
PNUSAL008415	SAMN16583316	PNUSAL000857	SAMN02950463
PNUSAL008399	SAMN16569104	PNUSAL008420	SAMN16583317
PNUSAL008568	SAMN16689233	PNUSAL001237	SAMN03265632
PNUSAL008138	SAMN16355300	PNUSAL001024	SAMN03093481
PNUSAL008397	SAMN16569147	PNUSAL001124	SAMN03153523
PNUSAL000741	SAMN02854691	PNUSAL000156	SAMN02351410
PNUSAL008418	SAMN16583318	PNUSAL000969	SAMN03067777
PNUSAL008567	SAMN17097836	PNUSAL008396	SAMN16569143
PNUSAL008564	SAMN16689231	PNUSAL008416	SAMN16583327
PNUSAL008276	SAMN16439299	PNUSAL008394	SAMN16569145
PNUSAL008404	SAMN16569112	PNUSAL008196	SAMN16392409

PNUSAL008412	SAMN16583319	PNUSAL000068	SAMN02265489
PNUSAL008197	SAMN16392406	PNUSAL008413	SAMN16583332
PNUSAL008565	SAMN16689248	PNUSAL001035	SAMN03093492
PNUSAL008395	SAMN16569097	PNUSAL001146	SAMN03178083
PNUSAL008275	SAMN16439300	PNUSAL001168	SAMN03198340
PNUSAL001758	SAMN04099861	PNUSAL001167	SAMN03198339
PNUSAL008401	SAMN16569107	PNUSAL008272	SAMN16439279
PNUSAL002183	SAMN05001507	PNUSAL008403	SAMN16569109
PNUSAL008566	SAMN16689234	PNUSAL008141	SAMN16355298
PNUSAL000024	SAMN02265455	PNUSAL008194	SAMN16392405
PNUSAL008422	SAMN16583330	PNUSAL001025	SAMN03093482
PNUSAL001719	SAMN04090018	PNUSAL008140	SAMN16355299
PNUSAL008195	SAMN16392403	PNUSAL001127	SAMN03153526

Table S3. The accession numbers of 73 *L. monocytogenes* strains

Number	Biosample No.	PN ID	Isolate No.	Accession No.
1	SAMN16912425	PNUSAL008695		PDT000897850.1
2	SAMN10341963	PNUSAL004479		PDT000398721.1
3	SAMN17977670	PNUSAL009095		PDT000967626.1
4	SAMN15734918	PNUSAL007665		PDT000802489.1
5	SAMN15338716	PNUSAL007447		PDT000769782.1
6	SAMN14306102	PNUSAL006998		PDT000706050.1
7	SAMN07965052		C2017017748	PDT000260500.2
8	SAMN14534623	PNUSAL007132	C2020005203	PDT000718710.1
9	SAMN14534621	PNUSAL007136	C2020005207	PDT000718676.1
10	SAMN05179386		C2010000503	PDT000154661.2
11	SAMN12912879	PNUSAL005887		PDT000600710.1
12	SAMN05179388		C2010000507	PDT000149268.2
13	SAMN14534624	PNUSAL007131	C2020005202	PDT000718674.1
14	SAMN09991607		C2018013398	PDT000376795.1
15	SAMN14534619	PNUSAL007135	C2020005206	PDT000718709.1
16	SAMN14306168	PNUSAL007023		PDT000706052.1
17	SAMN05179390		C2010001336	PDT000154658.2
18	SAMN14534622	PNUSAL007137	C2020005208	PDT000718711.1
19	SAMN05179385		C2010000502	PDT000154663.2
20	SAMN14534620	PNUSAL007129	C2020005200	PDT000718673.1
21	SAMN10986845	PNUSAL004717		PDT000468440.1
22	SAMN14600349	PNUSAL007234	C2020005211	PDT000722360.1
23	SAMN14534617	PNUSAL007133	C2020005204	PDT000718713.1
24	SAMN13811298	PNUSAL006748		PDT000658601.1
25	SAMN08111027	PNUSAL003601		PDT000267905.2
26	SAMN14534618	PNUSAL007130	C2020005201	PDT000718672.1
27	SAMN14380328	PNUSAL007045	C2020004501	PDT000709532.1
28	SAMN12912884	PNUSAL005854		PDT000600714.1
29	SAMN14534626	PNUSAL007128	C2020005199	PDT000718708.1
30	SAMN14534616	PNUSAL007134	C2020005205	PDT000718712.1
31	SAMN09499895	PNUSAL004043		PDT000337511.1
32	SAMN14381860	PNUSAL007047	C2020004502	PDT000709544.1
33	SAMN18129016	PNUSAL009267		PDT000977353.1
34	SAMN07796271	PNUSAL003463		PDT000253476.2
35	SAMN14600347	PNUSAL007233	C2020005210	PDT000722361.1
36	SAMN14381858	PNUSAL007046	C2020004500	PDT000709545.1
37	SAMN05179387		C2010000505	PDT000148618.2
38	SAMN05179383		C2009048931	PDT000137422.2
39	SAMN14534625	PNUSAL007138	C2020005209	PDT000718675.1
40	SAMN15447707	PNUSAL007519		PDT000781800.1

41	SAMN09500706	PNUSAL004017		PDT000337512.1
42	SAMN12912877	PNUSAL005856		PDT000600976.1
43	SAMN10715328	PNUSAL004564		PDT000432243.1
44	SAMN15969419	PNUSAL007886		PDT000828401.1
45	SAMN12912866	PNUSAL005996		PDT000600708.1
46	SAMN14367345	PNUSAL007044		PDT000708719.1
47	SAMN16248722	PNUSAL008067		PDT000843571.1
48	SAMN07757677	PNUSAL003451		PDT000250268.2
49	SAMN09690772	PNUSAL004087		PDT000351603.1
50	SAMN08712058	PNUSAL003847		PDT000294491.2
51	SAMN05179384		C2009048932	PDT000154662.2
52	SAMN14169866	PNUSAL006987		PDT000690044.1
53	SAMN07796272	PNUSAL003467		PDT000253477.2
54	SAMN05179389		C2010000509	PDT000148617.2
55	SAMN12822639	PNUSAL005846		PDT000591958.1
56	SAMN12667724	PNUSAL005336		PDT000577461.1
57	SAMN07982472	PNUSAL003551		PDT000263679.2
58	SAMN10149541	PNUSAL004399		PDT000387126.1
59	SAMN08647053	PNUSAL003826		PDT000293142.2
60	SAMN07358447	PNUSAL003140		PDT000225636.2
61	SAMN10231307	PNUSAL004427		PDT000390365.1
62	SAMN07812418	PNUSAL003468		PDT000257196.2
63	SAMN15147189	PNUSAL007403		PDT000760491.1
64	SAMN07757678	PNUSAL003452		PDT000250264.3
65	SAMN07982471	PNUSAL003550		PDT000263665.2
66	SAMN07585812	PNUSAL003266		PDT000259291.2
67	SAMN14426879	PNUSAL007071		PDT000712342.1
68	SAMN12822638	PNUSAL005847		PDT000591953.1
69	SAMN08056746	PNUSAL003591		PDT000267448.2
70	SAMN07457483	PNUSAL003228		PDT000233012.2
71	SAMN16439280	PNUSAL008274		PDT000861756.1
72	SAMN07457482	PNUSAL003227		PDT000233013.2
73	SAMN16439278	PNUSAL008273		PDT000861764.1

Table S4. Primers used in this study

Name	Sequence (5'-3')
rmlD-SalI-A	AAGTCGACGTAACAGGTGGGGCAGGTTT
rmlD-B	GCTGACCATTTGCTCCTGTTA
rmlD-C	TAACAGGAGCAAATGGTCAGCGGCAAGATGCTTTAGTTCGTTTT
rmlD,operon-NcoI-D	AACCATGGGCCAGCGTAAACACCACTTTA
Clon-rmlD-SalI-F	GTCGACCGCGCAAAGTTGGGTGAATAA
Clon-rmlD-NotI-R	CCATGGGGCTCCTTTTGTTCATAGAGTAATT
operon-SalI-A	AAGTCGACAGACACATCCAAACGCGAGTATAA
operon-B	CCTTTTAATGAATTGAGTACTCTATC
operon-C	GATAGAGTACTCAATTCATTAAAAGGGGCAAGATGCTTTAGTTCGTTTT
pHoss1-con-F	CTAACATTGCTAGTGTGAAGAAAAT
pHoss1-con-R	CCGGAAGCGAGAAGAATCATAA
